# Low RYR2 Level Relates to Poor Prognosis of Patients With Lung Adenocarcinoma by Promoting Tumor Cell Proliferation and Inhibiting Immune Cell Infiltration

**DOI:** 10.1002/bab.2759

**Published:** 2025-04-08

**Authors:** Tao Wang, Baozhen Wang, Zhongting Lu, Tao Li

**Affiliations:** ^1^ Department of Surgical Oncology II General Hospital of Ningxia Medical University Yinchuan Ningxia China

**Keywords:** lung adenocarcinoma, oxygen consumption rate, ryanodine receptor type 2, tumor infiltrated immune cells

## Abstract

Ryanodine receptor type 2 (RYR2) is a large calcium channel that has been identified as one of the most frequently mutated genes in lung adenocarcinoma (LUAD). Despite its potential significance, the role of RYR2 in LUAD remains poorly understood. In this study, we obtained transcriptomic data (normal *n* = 59, tumor *n* = 541) from TCGA portal and RYR2 protein abundance data from cProSite, which includes 86 normal and 91 tumor samples. Additionally, we assembled a cohort of 38 patients with LUAD and collected paired tumor and adjacent non‐tumor control samples. To investigate the functional impact of RYR2, we employed 3‐(4,5‐dimethylthiazol‐2‐yl)‐2,5‐diphenyltetrazolium bromide (MTT) assay and flow cytometry analysis to assess cell viability and apoptosis, respectively. While mitochondria function was evaluated via measuring oxygen consumption rate. The relationship between RYR2 expression level and immune cell infiltration was analyzed by immunohistochemistry and flow cytometry analysis. Furthermore, RT‐qPCR and enzyme‐linked immunosorbent assay were used to quantify the expression levels of CCL14 and CXCL12. Our findings demonstrated that both the mRNA and protein levels of RYR2 were significantly downregulated in LUAD samples, and lower RYR2 levels are associated with the poor patient prognosis. Overexpression of RYR2 in A549 and H1299 cells resulted in impaired mitochondrial function, decreased cell viability, and increased apoptosis. Notably, RYR2 levels exhibited a negative correlation with tumor purity, and tumors with lower RYR2 levels showed diminished infiltration of T cells and dendritic cells. Knockdown of RYR2 in LUAD cells inhibited the production of chemokines, particularly CCL14 and CXCL12. In conclusion, our study reveals that RYR2 functions as a tumor suppressor in LUAD by inducing mitochondrial dysfunction and promoting immune cell infiltration.

## 1 Introduction

Lung adenocarcinoma (LUAD), accounting for approximately 40% of all lung cancer cases, is the most prevalent subtype of lung cancer [1]. The identification of dysregulated or mutated oncogenes that promote tumor growth has paved the way for the development of more effective and less toxic targeted therapies for patients with LUAD [2]. However, the frequent emergence of resistance and subsequent relapses remain significant challenges, often resulting in poor prognosis of those affected. Thus, there is an urgent need to identify new therapeutic targets for LUAD treatment.

Calcium plays a vital regulatory role in various biological processes, including the initiation and progression of cancers [3]. In LUAD, calcium signaling is particularly important, with specific Ca^2^⁺ channels and binding proteins exhibiting alterations linked to dysregulated cell proliferation, apoptosis, and metastasis [4, 5]. Additionally, the morphology and function of mitochondria are sensitive to intracellular calcium levels that are essential for cellular energy production [6]. Disruption of Ca^2+^ homeostasis in the endoplasmic reticulum has also been linked to the development of drug resistance in LUAD cells [4]. Therefore, understanding the regulation of Ca^2+^ homeostasis is crucial for elucidating the molecular mechanisms underlying lung cancer development and could facilitate the creation of new therapies targeting calcium signaling.

Ryanodine receptor type 2 (RYR2) is a large calcium channel mainly located in the cardiac sarcolemma reticulum [7]. Advancements in high‐throughput sequencing technology have revealed that RYR2 is one of the most frequently mutated genes in various cancers [8, 9, 10, 11]. However, research on the role of RYR2 in cancer is still in its early stages, and some findings are contradictory [12]. For example, Schmitt et al. analyzed whole‐exome sequencing, global DNA methylation, and gene expression profiling data, identifying somatic mutations and epigenetic silencing of RYR2 as common occurrences in head and neck squamous cell carcinoma [13]. In contrast, Chen et al. suggested that RyR2 may act as an oncogene with its promoting metastasis of colorectal cancer cells via the ROS/BACH1 axis [14]. In LUAD, RYR2 is associated with a high tumor mutation burden and serves as an independent risk factor for patient prognosis [15]. Nevertheless, the underlying mechanisms remain unclear.

In this study, we examined the expression levels of RYR2 in tumor and adjacent non‐tumor samples from patients with LUAD. We further evaluated the functional roles of RYR2 in tumor cells and tumor microenvironments to elucidate its potential impact on LUAD progression and therapy.

## 2 Materials and Methods

### 2.1 Databases and Patients

This study included clinical information and gene expression profiling data of 541 patients with LUAD from TCGA that was downloaded from cBioPortal database (https://www.cbioportal.org/).

Protein levels of RYR2 in 91 LUAD tumors and 86 paired adjacent non‐tumor control samples were analyzed using cProtSite (https://cprosite.ccr.cancer.gov/).

Thirty‐eight pairs of LUAD tissues and corresponding normal tissues were collected from the patients during surgeries at General Hospital of Ningxia Medical University between May 2024 and September 2024. Table 1 shows the main clinical characteristics of all patients. All participants were informed of the purpose and the details of this study, and they provided the written informed consent. The authors had access to identifiable information about individual participants after the data collection process.

**TABLE 1:** Clinical characteristics of patients with lung adenocarcinoma (LUAD).

Clinicopathological features	*N*
Gender	
Male	23
Female	15
Age (years)	
< 65	18
≥65	20
Smoking status	
Nonsmokers	22
Smokers	16
N‐stage (lymph node metastasis)	
N0	9
N1	18
N2	9
N3	2
Clinical TNM‐stage	
I	5
II	24
III	19

### 2.2 Cell Culture

Human LUAD cell lines H1299 (cat.no. CRL‐5803) and A549 (cat.no. CRM‐CCL‐185) were commercially purchased from the American Type Culture Collection (ATCC). The cells were cultured in Dulbecco's modified Eagle's medium (DMEM) supplemented with 10% fetal calf serum (Hyclone), 20 mmol/L HEPES, 100 IU/mL penicillin, and 100 µg/mL streptomycin.

### 2.3 RNA Extraction

Triol reagent (Invitrogen, Carlsbad, CA, USA) was used to extract total RNA from tissue and cell samples. The concentration and purity of the obtained RNA were assessed with an ND‐1000 spectrophotometer (Nanodrop Technologies, Wilmington, DE, USA). Only samples with absorbance ratios 260/280 nm of ∼2.0 and 260/230 nm of 1.9–2.2 were included in the study.

### 2.4 Quantitative RT‐PCR

The expression levels of candidate genes were quantified using RT‐qPCR with SYBR Green real‐time PCR master mix (Thermo Fisher Scientific), using β‐actin as the loading control. Each sample within each group was measured in triplicate, and the experiment was conducted at least three times. The relative levels of the target genes were determined using the −ΔΔCt method. The primer sequences are listed in Table 2.

**TABLE 2:** Primers for RT‐qPCR.

Gene name	Accession number	Primer name	Sequence	Position
*CXCL12*	NM_199168	CXCL12‐F	GCACTTTCACTCTCCGTCAG	Exon 1 (4–23)
		CXCL12‐R	CACGACCTTGGCGTTCAT	Exon 1 (93–110)
*CCL14*	NM_032962	CCL14‐F	GTGCTGCTTCACCTACACTAC	Exon 3 (230–251)
		CCL14‐R	CACTGGCTGTTGGTCTCATAG	Exon 3 (285–306)
*ACTB*	NM_001101.5	ACTB‐F	GGCATGGGTCAGAAGGATT	Exon 3 (221–240)
		ACTB‐R	AAGGTGTGGTGCCAGATTT	Exon 3 (336–355)

### 2.5 Vector Construction and Electroporation

We amplified two segments of the RYR2 coding sequence separately by PCR using cDNA of AC16 cells as the template that was purchased from SUNNCELL company. The two segments of RYR2 were sequentially cloned into pcDNA3.1(+) vector (Figure S1) between Nhe I and Xho I sites to assemble the full‐length RYR2 expression sequence and generate the RYR2 overexpression vector. Primer sequences were provided in Table 3. The RYR2 overexpression vector is designated as pcDNA3.1‐RYR2. This vector, along with an empty control vector, was transfected into A549 and H1299 cells using electrotransfection with a 4D‐Nucleofector X Unit (Lonza, Tampa, FL, USA) and a P3 Primary Cell 4D‐Nucleofector X Kit (Lonza, Tampa, FL, USA) following the manufacturer's instructions.

**TABLE 3:** Primers for ryanodine receptor type 2 (RYR2) cloning.

Primer name	Sequence
RYR2‐fragment1‐Nhe‐F	CCGCTAGCATGGCCGATGGGGGCGAGGGCGAAGAC
RYR2‐fragment1‐Kpn‐R	GCAAAGGTACCGATTGAGGGCCAAGGCCATGTC
RYR2‐fragment2‐Kpn‐F	AATCGGTACCTTTGCACAGCCGTCTTGCCATTG
RYR2‐fragment2‐Xho‐R	CCCTCGAGATTTAGCTGGTCTTCATACTGTTTCCGGAAGC

### 2.6 Knockdown RYR2 Using Small Interfering RNAs (siRNAs)

Two siRNAs targeting RYR2 and negative control siRNA (Table 4) were synthesized by Integrated DNA Technologies, Inc. A549 and H1299 were seeded into 24‐well plate and allowed to attach overnight. Diluted the siRNA in 250 µL of Opti‐MEM or serum‐free medium to a final concentration of 100 nM. In a separate tube, dilute the 20 µL of lipofectamine 2000 in 250 µL of Opti‐MEM and incubate for 5 min at room temperature. Combine the diluted siRNA with the diluted transfection reagent. Mix gently and incubate for 20 min at room temperature to allow complex formation. Remove the growth medium from the cells and add 500 µL of Opti‐MEM to each well. Carefully added the siRNA‐transfection reagent complexes dropwise to the cells. Gently swirled the plate to ensure even distribution. Incubate the cells at 37°C with 5% CO_2_ for 6 h and then removed the transfection reagents. The cells were cultured in growth medium from another 48 h and then subjected other experiments.

**TABLE 4:** Sequences for small interfering RNAs (siRNAs).

Name	Sequence
Si‐control	UUCUCCGAACGUGUCACGUTT
	ACGUGACACGUUCGGAGAATT
Si‐RYR2‐1	CAGUCAUCAAUGGAUUCUGAA
	UUCAGAAUCCAUUGAUGACUG
Si‐RYR2‐2	AAAAUCAACAGCAUUUACCTT
	AAGGUAAAUGCUGUUGAUUUU

Abbreviation: RYR2, ryanodine receptor type 2.

### 2.7 Cell Viability Analysis

Cell proliferation was estimated by the 3‐(4,5‐dimethylthiazol‐2‐yl)‐2,5‐diphenyltetrazolium bromide (MTT) assay. Cells were seeded in wells of 96‐well plates at low density (2 × 10^3^) in DMEM medium and allowed to attach overnight. The cells were then transfected with siRNA targeting RYR2 or RYR2 overexpression vector. There were three wells in each group. Twenty microliters of MTT (5 mg/mL) (Sigma‐Aldrich) were added to each well 48 h after transfection, and the cells were incubated for a further 4 h. The absorbance was recorded at 570 nm with a 96‐well plate reader after addition of dimethyl sulfoxide (DMSO):

$$\begin{array}{ccc} \mathrm{Relative}\mathrm{cell}\mathrm{viability} & = & \mathrm{absorbance}\mathrm{of}\mathrm{treated}\mathrm{cells}/\\ & & \;\mathrm{absorbance}\mathrm{of}\mathrm{control}\mathrm{cells} \end{array}$$

### 2.8 Colony Formation Analysis

Cells were seeded at a low density (1000 cells/well) in 6‐well plates. Use complete growth medium and incubate cells overnight at 37°C with 5% CO_2_ for 10 days. The medium was refreshed every 3 days. Cells were fixed by adding 4% paraformaldehyde to each well and incubating for 10 min at room temperature. The colonies were stained with 0.5% crystal violet solution and then counted.

### 2.9 Immunoblotting

Proteins were extracted by using RIPA buffer (Thermo Scientific, Waltham, MA, USA) and then quantified by using the Bradford protein assay kit (Bio‐Rad, Hercules, CA, USA) according to the manufacturer's instructions. The proteins were denatured by boiling in sample buffer containing sodium dodecyl sulfate/β‐mercaptoethanol. Subsequently, 10 µg of each sample was loaded into each lane of 12% polyacrylamide gels, and the proteins were separated by electrophoresis. The proteins were transferred to a polyvinylidene fluoride (PVDF) membrane (0.45 µm pore size) (Amersham Pharmacia Biotech, St. Albans, Herts, UK) via electrophoretic transfer. The membrane was blocked by 5% non‐fat milk and then incubated with the primary antibody for RYR2 (cat no. ab196355, Abcam, 1:500 dilution) or GAPDH (cat no. 2118, Cell Signaling Technology, 1:1000 dilution) overnight at 4°C. The membrane was washed three times with Tris‐buffered saline with Tween‐20 (TBST) (20 mM Tris, 150 mM NaCl, Tween‐20 detergent 0.1%, pH 7.4) for 10 min each to remove unbound primary antibody. The membrane was incubated with the horseradish peroxidase (HRP) conjugated secondary antibody for 1 h at room temperature. The membrane was washed with TBST for another three times, and the detection was performed using chemiluminescence with a SuperSignal West Femto Maximum Sensitivity Substrate kit (Thermo Fisher Scientific), employing GAPDH as a loading control.

### 2.10 Immunohistochemistry (IHC)

Paraffin‐infiltrated samples were sectioned using a microtome to a 4 µm thickness and placed on positively charged slides. Baked slides at 60°C for 2 h to help the sample to adhere. Paraffin‐embedded sections were first deparaffinized using xylene and gradient ethanol. Boil slides in antigen retrieval buffer (10 mM sodium citrate, 0.05% Tween 20, pH 6.0) and maintain at approximately 98°C for 20 min. The slides were cooled into room temperature and then incubated overnight at 4°C with the diluted primary antibody. The sections were washed by TBST (20 mM Tris, 150 mM NaCl, Tween‐20 detergent 0.1%, pH 7.4) for three times and then were subsequently incubated with the HRP‐conjugated secondary antibody. After another three washes with TBST, the signals were detected using a diaminobenzidine (DAB) substrate kit (cat.no. 8059, Cell Signaling Technology) according to the manufacturer's instructions. Images were captured using microscopy, and an H‐score ranging from 0 to 300 was calculated by multiplying the staining intensity by the percentage of tumor cells exhibiting positive staining. The accuracy of automated measurements was verified by two independent investigators.

#### 2.10.1 Antibody Information

Rabbit anti‐RYR2 polyclonal antibody (cat.no. ab196355, Abcam, 1:200 dilution), Rabbit anti‐CD4 monoclonal antibody (cat.no. 25229, Cell Signaling Technology, 1:200 dilution), Rabbit anti‐CD8 monoclonal antibody (cat.no. 98941, Cell Signaling Technology, 1:200 dilution), Rabbit anti‐CD11c monoclonal antibody (cat.no. 97585, Cell Signaling Technology, 1:200 dilution), HRP‐conjugated goat anti‐rabbit secondary antibody (cat.no. 7074, Cell Signaling Technology, 1:500 dilution).

### 2.11 Flow Cytometry Analysis

Prepared the antibody cocktail by diluting a panel of fluorochromes conjugated monoclonal antibodies. These antibodies include a pan leucocyte marker, CD45; lineage markers (CD3, CD19, CD20, CD14, and CD56) as well as HLA‐DR to identify the CD45+, lineage‐, HLA‐DR+ dendritic cell populations. Cells were washed by PBS for three times, and the cell number was adjusted to a concentration of 5 × 10^6^ cells/mL in ice‐cold fluorescence‐activated cell sorting (FACS) buffer (PBS supplemented with 1% BSA and 0.1% sodium azide). Add 100 µL of cell suspension to each tube and mix with the antibody cocktail. After 30 min incubation at 4°C in dark, the cells were washed by FACS buffer for three times. Resuspended the cells in 500 µL ice‐cold FACS buffer. The cells were analyzed using a FACSCanto II flow cytometer. The results were processed with FlowJo software (v10.4.1) (Tree Star Inc.).

#### 2.11.1 Antibody Information

Mouse Anti‐Human CD45 antibody (cat.no. 340953, BD, 1:200 dilution),

FITC Mouse Anti‐Human CD3 antibody (cat.no. 561806, BD, 1:500 dilution),

FITC Mouse Anti‐Human CD19 antibody (cat.no. 560994, BD, 1:500 dilution),

FITC Mouse Anti‐Human CD20 antibody (cat.no. 560962, BD, 1:500 dilution),

FITC Mouse Anti‐Human CD14 antibody (cat.no. 561712, BD, 1:500 dilution),

FITC Mouse anti‐Human CD56 antibody (cat.no. 562794, BD, 1:500 dilution),

PE Mouse Anti‐Human HLA‐DR antibody (cat.no. 560943, BD, 1:500 dilution),

V450 Mouse Anti‐Human CD4 antibody (cat.no. 560811, BD, 1:500 dilution),

APC Mouse Anti‐Human CD8 (cat.no. 566852, BD, 1:500 dilution).

Apoptotic cell analysis was used Annexin V‐FITC Early Apoptosis Detection Kit (cat.no. 6592, Cell Signaling Technology) following the manufacturer's instructions. Briefly, cells were washed with ice‐cold PBS for three times and then resuspended at 10^6^ cells/mL with 1× Annexin V Binding Buffer. Added 1 µL Annexin V‐FITC conjugate and 12.5 µL propidium iodide (PI) solution to each 96 µL cell suspension. Incubated the cells for 10 min on ice in the dark. Diluted the cell suspension to a final volume of 250 µL per assay with ice‐cold, 1× Annexin V Binding Buffer and then subjected to flow cytometry analysis.

### 2.12 Oxygen Consumption Assay

The mitochondria activity was assessed using a Seahorse XF24 Analyzer and a Seahorse XF Cell Mito Stress Test Assay kit (Agilent Technologies). The oxygen consumption rate (OCR) was measured to evaluate cell mitochondrial functionality, following the manufacturer's instructions. In briefly, 8 × 10^4^ cells were seeded into a 24‐well plate and allowed to adhere overnight in serum‐reduced medium (1% fetal calf serum). Before OCR detection, the serum‐reduced medium was replaced by complete growth medium and cultured for 2 h. The OCR detection was performed with sequential injections of oligomycin, carbonyl cyanide *p*‐trifluoromethoxyphenylhydrazone (FCCP), and a combination of rotenone and antimycin A.

### 2.13 Statistical Analysis

Data were conducted using R (version 4.2.2) via RStudio (Desktop version, 2022.12.0+353). Data from more than two groups were analyzed using one‐way ANOVA method through multiple comparison test. Kaplan–Meier analysis with log‐rank test was used to compare the survival data from patients or mice. A *p* value less than 0.05 was considered to indicate statistical significance.

## 3 Results

### 3.1 RYR2 Expression Is Reduced in Patients With LUAD and Correlates With Poor Prognosis

**FIGURE 1:**
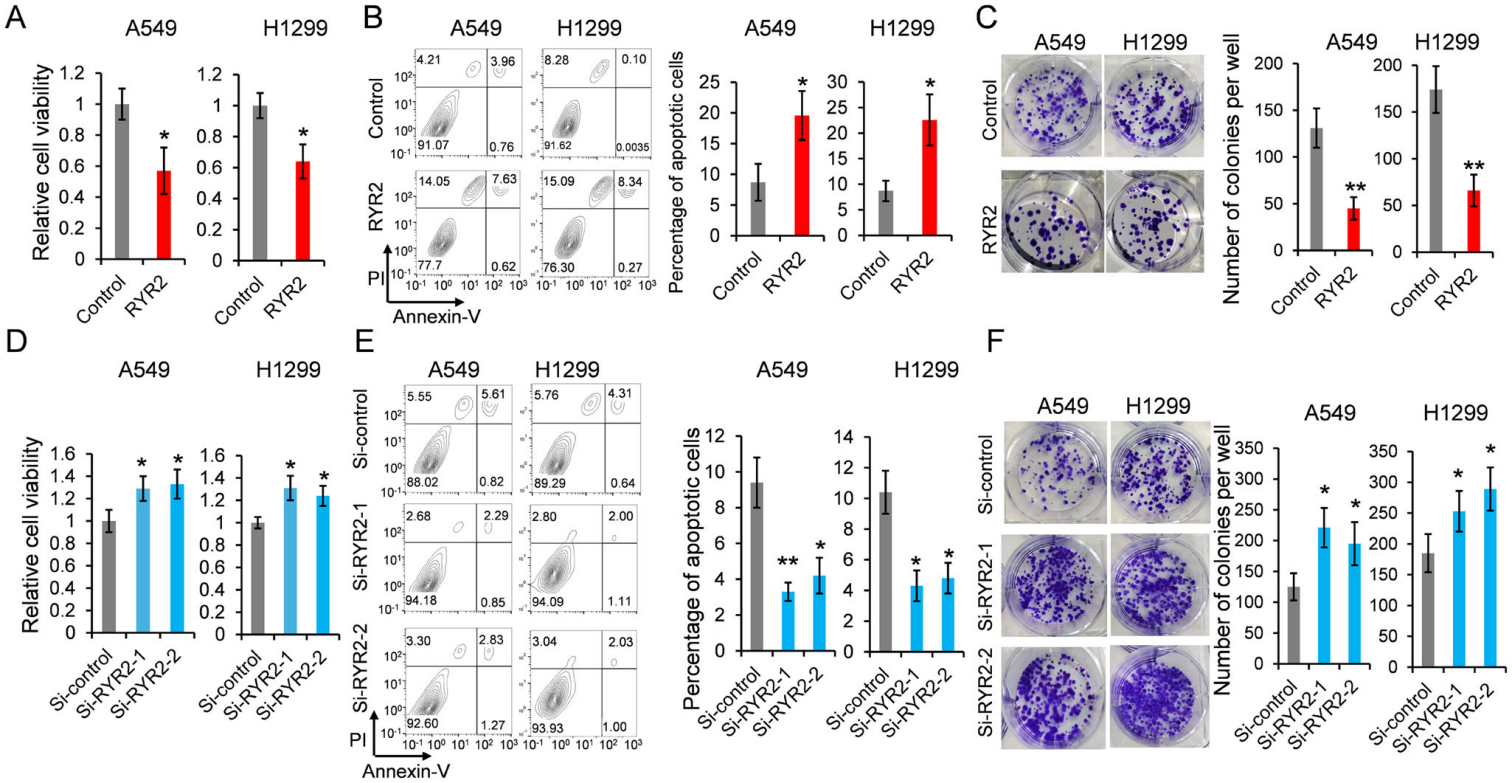
**RYR2 controls viability and apoptosis of LUAD cells**. RYR2 was overexpressed in A549 and H1299 cells. The cell viability was detected by MTT assay (A). Apoptotic cells were counted by flow cytometry analysis (B). RYR2 was knocked down by using RYR2 specific siRNAs. Colony formation assay was used to evaluate the clonogenic potential of cells (C). SiRNA targeting RYR2 was transfected into A549 and H1299 cells. The cell viability was detected by MTT assay (D). Apoptotic cells were counted by flow cytometry analysis (E). Colony formation assay was used to evaluate the clonogenic potential of cells (F). RYR2, ryanodine receptor type 2. *p < 0.05, **p < 0.01.

To explore the roles of calcium channels in the LUAD, we analyzed the mutation frequencies of the inositol triphosphate receptors 1–3 (ITP3R1–3) and the ryanodine receptors 1–3 (RYR1‐3) in LUAD patients that were included in the TCGA project. We noticed that RYR2 is the most frequently mutated calcium channel coding gene (Figure S1A) and ranks the fifth most frequently (Figure S1B) mutated gene overall in all LUAD patients. Both mRNA (Figure 1SC) and protein (Figure S1D) levels of RYR2 are reduced in LUAD tumor samples compared to adjacent non‐tumor tissues. Furthermore, patients with lower RYR2 levels had shorter overall survival (Figure S1E). To validate these findings, we collected 38 tumors samples, and 38 paired adjacent non‐tumor controls. RT‐qPCR analysis confirmed that RYR2 expression was consistently reduced in all the tumors (Figure S1F). These data suggest that RYR2 dysregulation may play a critical role in LUAD progression.

### 3.2 RYR2 Inhibits Cell Proliferation, Promotes Apoptosis, and Impairs the Mitochondria Function in LUAD Cells

To explore the role of RYR2 in LUAD, RYR2 coding sequence was cloned into pcDNA3.1 vector (Figure S2). The RYR2 overexpression vector was transfected into A549 and H1299 cells (Figure S3A). MTT assay revealed that RYR2‐overexpressing cells exhibited reduced viability (Figure 1A), whereas flow cytometry analysis showed increased apoptosis (Figure 1B) and fewer colonies formed (Figure 1C).

**FIGURE 2:**
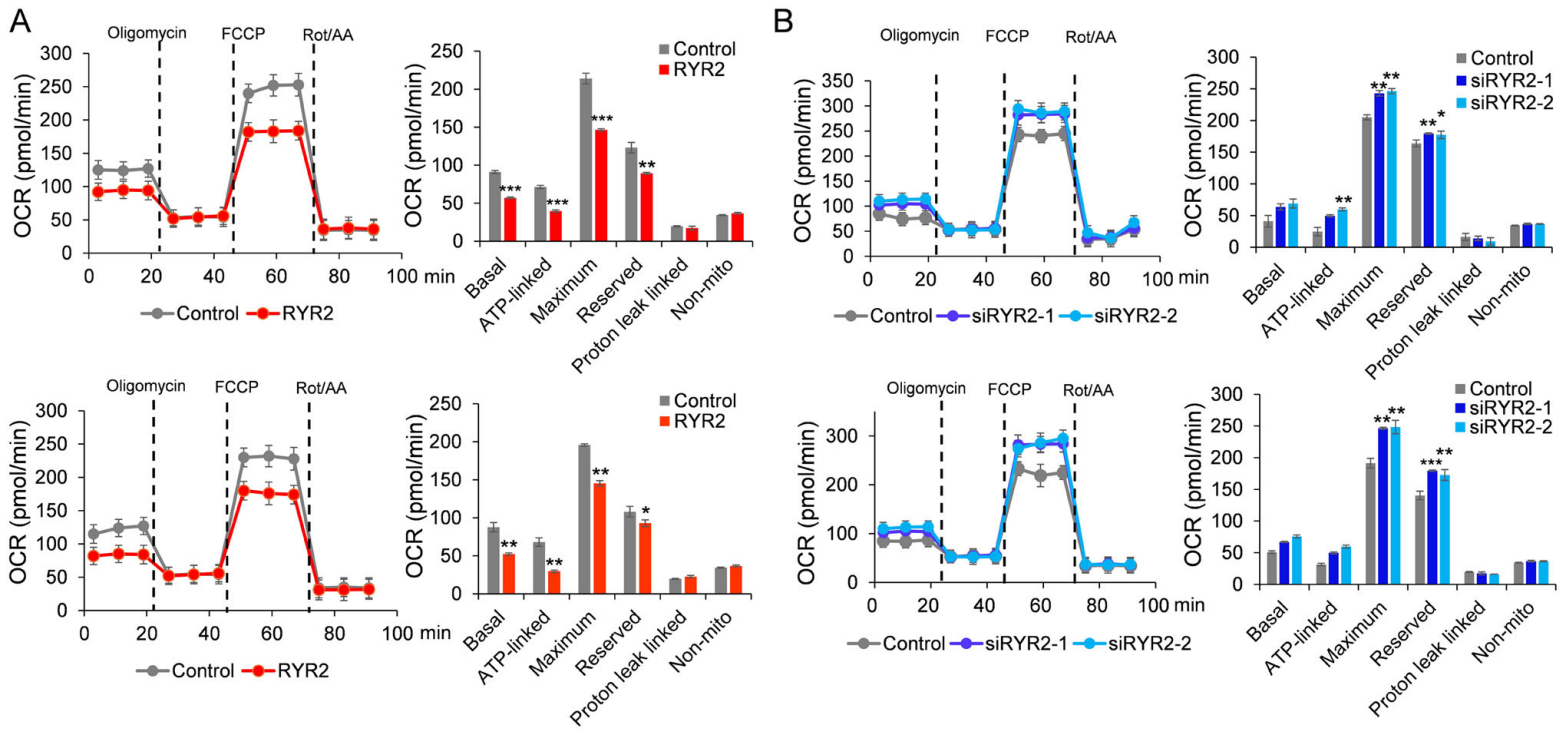
**RYR2 controls the oxygen consumption rate of LUAD cells**. The oxygen consumption rate of RYR2 overexpressed (A) or knocked down cells (B) was examined by a Seahorse XF24 Analyzer. OCR, oxygen consumption rate; RYR2, ryanodine receptor type 2. *p < 0.05, **p < 0.01, ***p < 0.001, ****p < 0.0001.*p < 0.05, **p < 0.01, ***p < 0.001.

Because calcium is an important regulatory factor that controls mitochondria homeostasis and function, we examined the OCRs to evaluate the impact of RYR2 on mitochondria in LUAD cells. As shown in Figure 2A, overexpression of RYR2 significantly repressed the OCR of LUAD cells, whereas RYR2 knockdown upregulated OCR (Figure 2B). These findings indicate that RYR2 modulates mitochondrial function in LUAD cells.

### 3.3 RYR2 Promotes the Infiltration of T Cells and Dendritic Cells in LUAD

**FIGURE 3:**
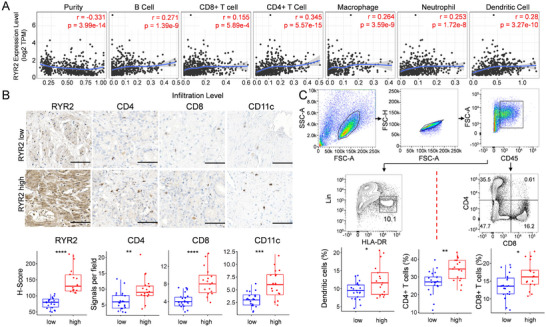
**RYR2 is negatively correlated with tumor purity**. (A) Tumor purity and immune cell infiltration analysis by using TIMER bioinformatics tool. (B) The levels of RYR2, CD4, CD8, and CD11c in LUAD tumors were detected by IHC. (C) Analysis of the proportions of tumor‐infiltrated dendritic cells (Lin‐, HLA‐DR+), CD4+ T cells, and CD8+ T cells. RYR2, ryanodine receptor type 2. *p < 0.05, **p < 0.01, ***p < 0.001, ****p < 0.0001.

LUAD can be classified into “hot” and “cold” tumors based on the level of immune cell infiltration that correlates with differential sensitivity to immunotherapy. To investigate the role of RYR2 in modulating tumor immune microenvironment, we analyzed the correlation of RYR2 level and immune cell infiltration using TIMER (https://cistrome.shinyapps.io/timer/). RYR2 level was negatively correlated with tumor purity and positively correlated with the infiltration of B cells, CD4+ T cells, CD8+ T cells, neutrophils, macrophages, and dendritic cells (Figure 3A). To confirm these findings, we performed IHC on tumor samples from 38 LUAD patients and stratified them into two groups based on RYR2 expression. Tumors with higher levels of RYR2 exhibited significantly increased infiltration of CD4+ T cells, CD8+ T cells, and CD11c+ dendritic cells (Figure 3B). Flow cytometry analysis further confirmed these observations (Figure 3C), suggesting that RYR2 promotes immune cell infiltration in LUAD.

### 3.4 RYR2 Positively Correlates With CCL4 and CXCL12 Levels in LUAD

**FIGURE 4:**
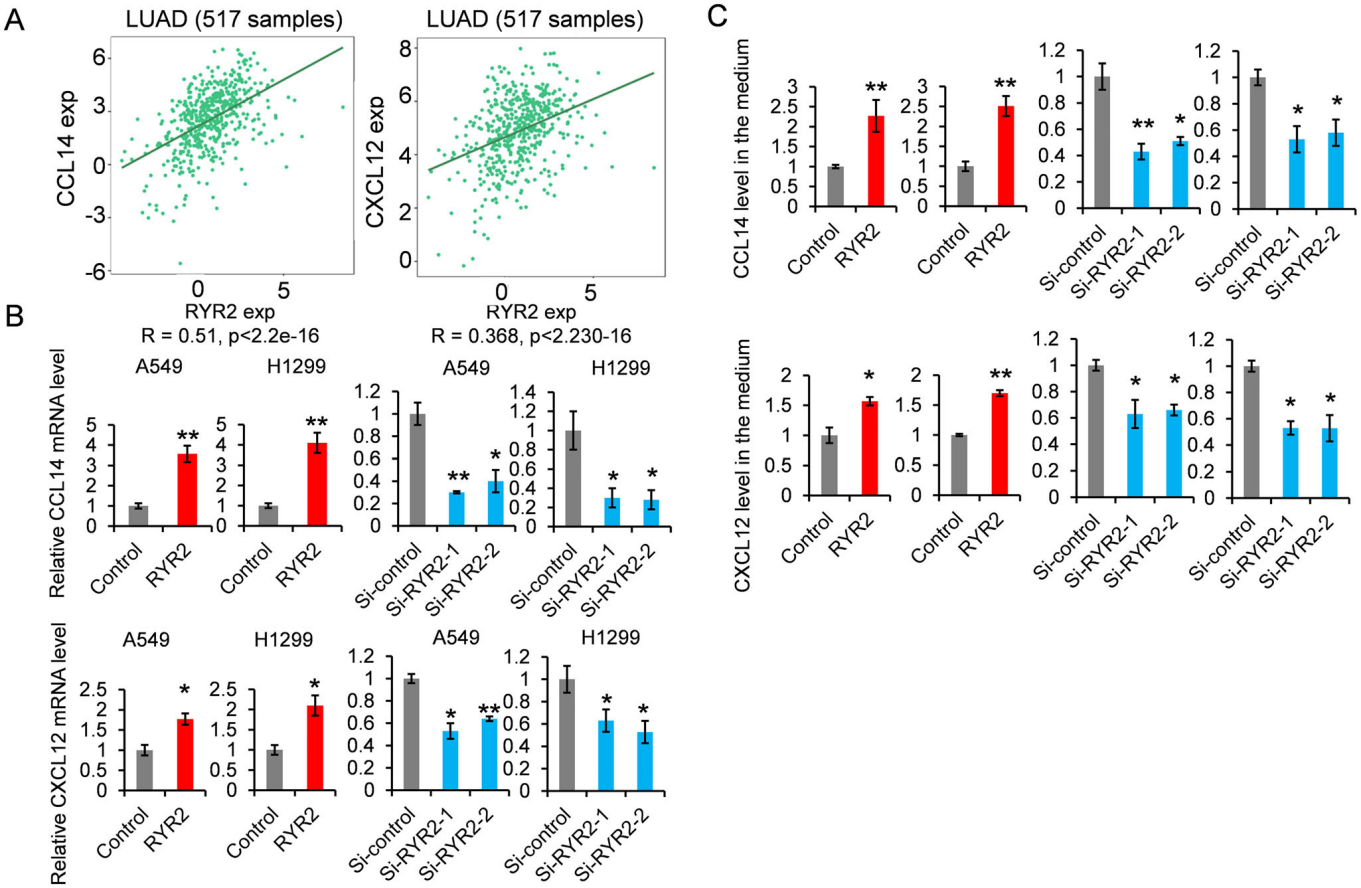
**RYR2 positively regulates the expression of CCL14 and CXCL12**. (A) Correlation analysis of RYR2 and chemokine expression in LUAD tumors by using TISIDB bioinformatics tool. CCL14 and CXCL12 are the two chemokines whose levels are strong positively correlated with RYR2. (B) The mRNA levels of CCL14 and CXCL12 in RYR2 overexpressed or knocked down cells were detected by RT‐qPCR. (C) The secreted CCL14 and CXCL12 in the medium were detected by ELISA. LUAD, lung adenocarcinoma; RYR2, ryanodine receptor type 2. *p < 0.05, **p < 0.01.

To explore the mechanism by which RYR2 promotes immune cell infiltration, we analyzed the correlation between RYR2 and chemokine expression using TISIDB (http://cis.hku.hk/TISIDB/index.php). RYR2 showed positive correlations with CCL4 and CXCL12 expression in LUAD (Figure 4A). Overexpression of RYR2 in A549 or H1299 cells significantly increased the mRNA levels of CCL4 and CXCL12 (Figure 4B) and enhanced their secretion into the medium (Figure 4C). These results indicated that RYR2 may promote immune cell infiltration by upregulating chemokine production.

**FIGURE 5:**
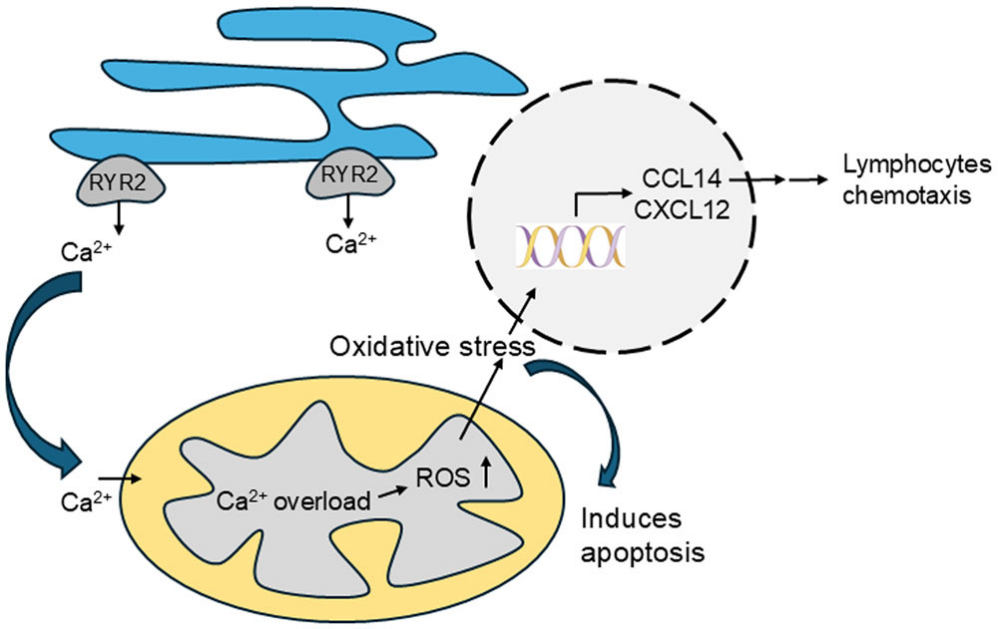
The schematic diagram of the role of RYR2 in lung adenocarcinoma. RYR2, ryanodine receptor type 2.

Finally, we proposed a working model for RYR2 in LUAD that upregulates RYR2 enhances the calcium transfer from ER to mitochondria, induces oxidative stress that triggers cell apoptosis, and produce chemokine (Figure 5).

## 4 Discussion

RYR2 is an important large calcium channel that is predominantly expressed in the heart with mutations linked to myopathies and arrhythmias [16, 17]. Recently, RYR2 was found to be one of the most mutated genes in multiple kinds of cancers [9, 10, 18]. However, the study of its assumed function in cancers is still in its initial stages. In this study, we analyzed the RYR2 mRNA and protein expression data from TCGA portal and cProSite, respectively. We found that RYR2 level is significantly reduced in LUAD samples. Importantly, lower RYR2 levels were associated with shorter overall survival, suggesting its potential as a prognostic biomarker for LUAD patients. To our knowledge, this is the first report highlighting the prognostic value of RYR2 in LUAD.

RYR2 regulates the intracellular calcium homeostasis that controls a wide variety of cellular processes, including the mitochondrial homeostasis and function. In ventricular myocytes, enhanced RyR2 activity alters sarcoplasmic reticulum (SR)‐mitochondrial Ca2+ transfer, elevates mito‐ROS emission, and finally results in self‐imposed exacerbation of SR Ca^2+^ leak [19]. In this study, we demonstrated for the first time that RYR2 overexpression impairs mitochondrial oxygen consumption, inhibits cell growth, and induces apoptosis in LUAD cells. The underlying mechanisms may be complex and relate to the mitochondrial ROS emission that needs to be further investigated.

Tumors are heterogeneous tissues that consist of various types of cells exhibiting multiple biological and genetic abnormalities. According to the distribution of cytotoxic immune cell distribution, tumors can be divided into “hot” and “cold” phenotypes [20]. In this study, we identified that tumors with higher RYR2 expressions had “hot” phenotype with reduced tumor purity and increased immune cell infiltration. RYR2‐overexpressing LUAD cells secreted elevated levels of chemokines, particularly CCL14 and CXCL12, that may facilitate immune cell chemotaxis.

There are limits of this study: (1) We observed disturbed mitochondrial function in RYR2 dysregulated cells, but the precise mechanism remains unclear; (2) although we identified RYR2 as a regulator of CCL14 and CXCL12 expression in LUAD cells, the molecular pathways underlying this regulation require further investigation.

In conclusion, our findings highlight RYR2 as a tumor suppressor in LUAD via inducing mitochondrial dysfunction and immune cell infiltration. These insights provide a foundation for future studies exploring RYR2 as a potential therapeutic target in LUAD.

## Author Contributions

**Tao Wang**: conceptualization, methodology, software, validation, formal analysis, investigation, resources, data curation, writing – original draft preparation, writing – review and editing, visualization. **Tao Li**: conceptualization, validation, formal analysis, resources, supervision, project administration, funding acquisition. **Baozhen Wang**: methodology, validation, investigation, data curation, visualization. **Zhongting Lu**: methodology, validation, investigation. All authors have read and agreed to the published version of the manuscript.

## Conflicts of Interest

The authors declare no conflicts of interest.

## Ethics Statement

The present study was approved by the Clinical Research Ethics Committee of General Hospital of Ningxia Medical University (approval no. KYLL‐2024‐0945).

## Consent

Written informed consent was obtained from all the participants.

## Supporting information



Figure S1. RYR2 is downregulated in LUAD tumors, and low RYR2 level relates to poor prognosis of LUAD patients.
(A) Genetic mutation frequency of RYR1‐3 and ITPR1‐3 in LUAD patients. (B) Distributions of most frequently mutated genes in LUAD patients. (C) RYR2 mRNA level is reduced in LUAD tumors. (D) RYR2 protein level is reduced in LUAD tumors. (E) Low RYR2 expression is related to shorter overall survival of LUAD patients. (F) Confirm the downregulation of RYR2 in 38 paired tumor and adjacent non‐tumor samples.

Figure S2 pcDNA3.1 vector map

Figure S3. RYR2 overexpression or knockdown in LUAD cells.
RYR2 overexpression vector or specific siRNAs targeting RYR2 were transiently transfected into A549 and H1299 cells. The RYR2 level was detected by immunoblotting.

## Data Availability

The authors confirm that the data supporting the findings of this study are available within the article and its supplementary materials.
