# Supplementary figures and images for: Low RYR2 Level Relates to Poor Prognosis of Patients With Lung Adenocarcinoma by Promoting Tumor Cell Proliferation and Inhibiting Immune Cell Infiltration
Source: Biotechnol Appl Biochem. 2025 Apr 8;72(6):1528–36. doi: 10.1002/bab.2759 (PMC12687703; doi:10.1002/bab.2759)

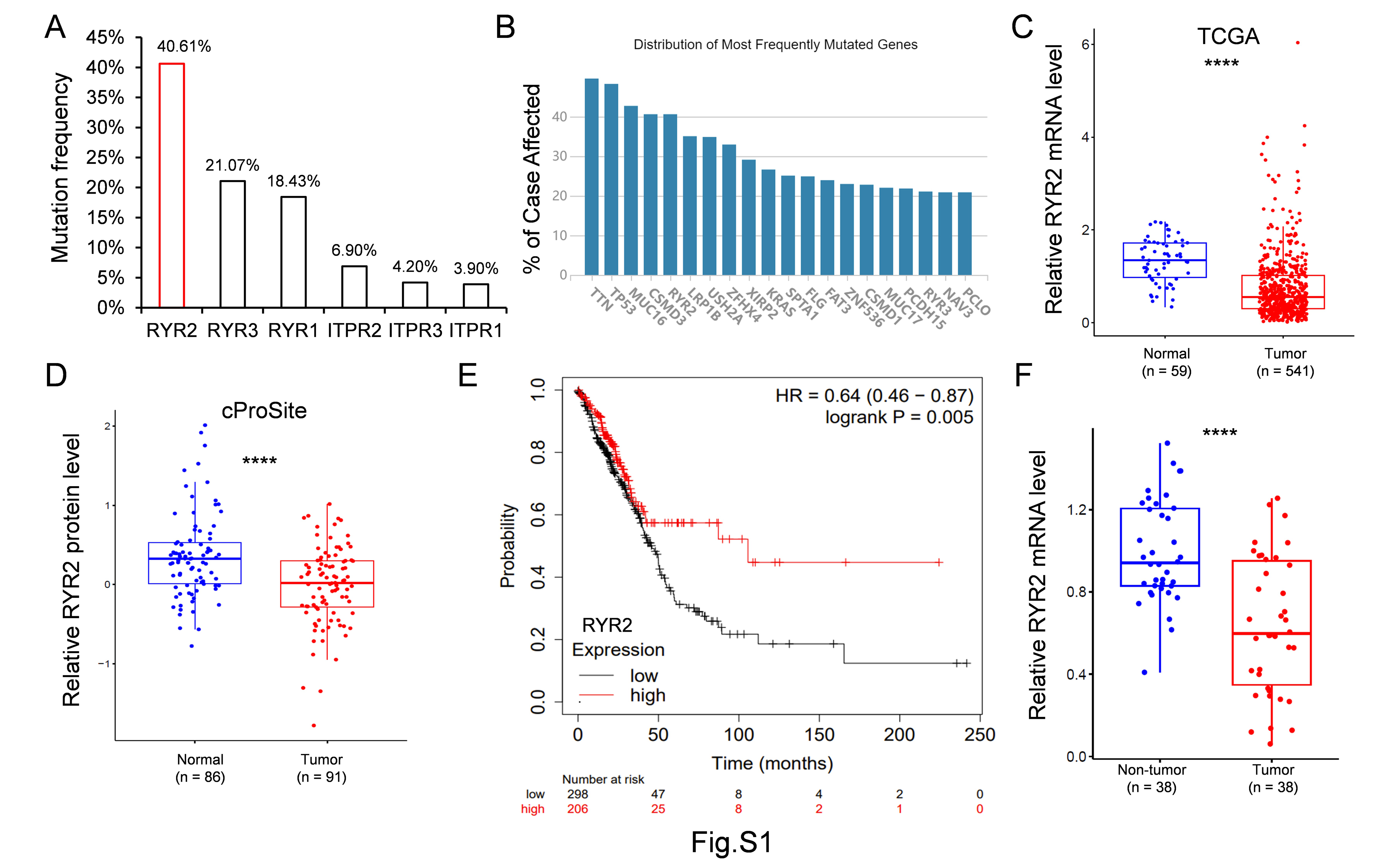

Supplement: Supplementary file 1 — Figure S1. RYR2 is downregulated in LUAD tumors, and low RYR2 level relates to poor prognosis of LUAD patients. (A) Genetic mutation frequency of RYR1‐3 and ITPR1‐3 in LUAD patients. (B) Distributions of most frequently mutated genes in LUAD patients. (C) RYR2 mRNA level is reduced in LUAD tumors. (D) RYR2 protein level is reduced in LUAD tumors. (E) Low RYR2 expression is related to shorter overall survival of LUAD patients. (F) Confirm the downregulation of RYR2 in 38 paired tumor and adjacent non‐tumor samples. [file BAB-72-1528-s002.tif]

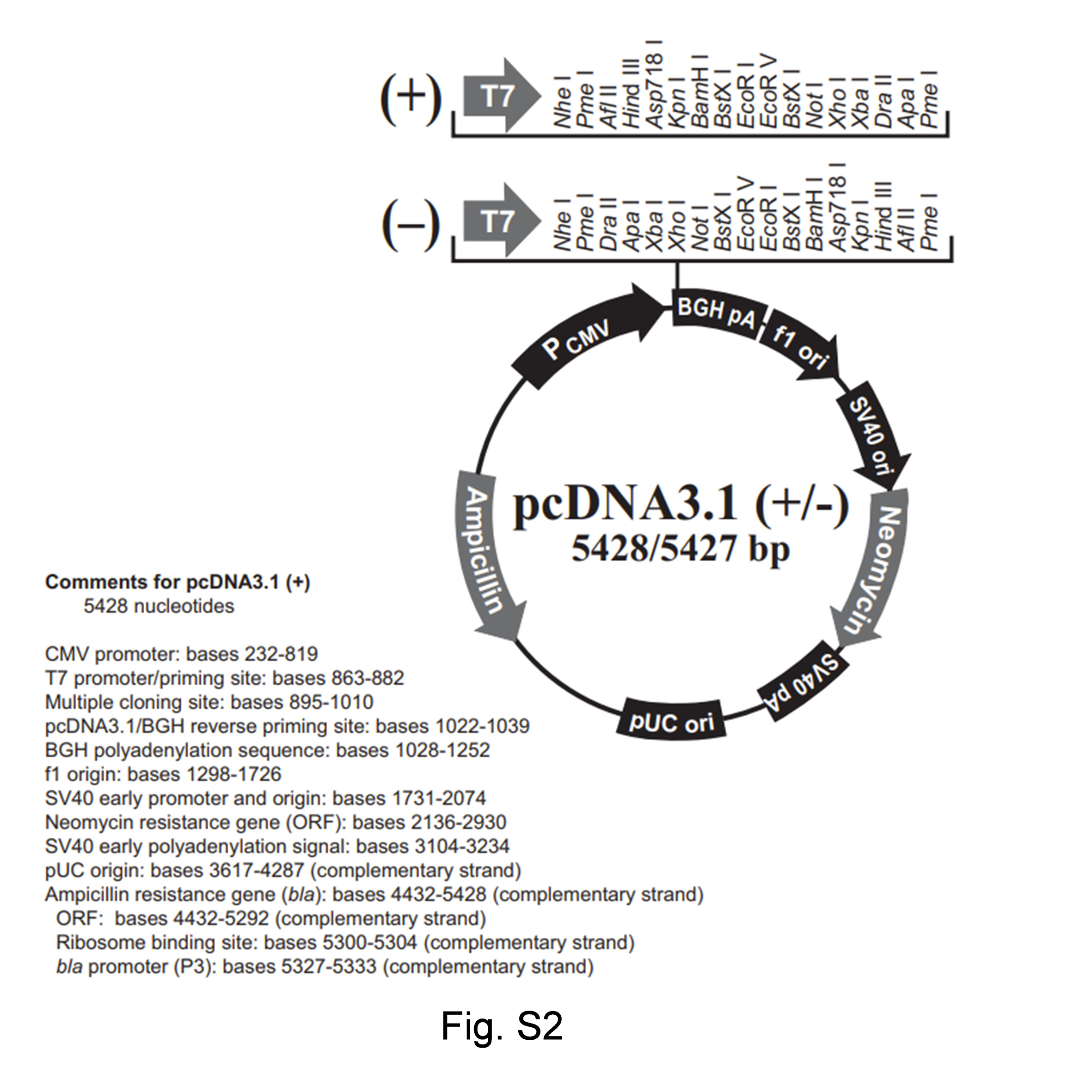

Supplement: Supplementary file 2 — Figure S2 pcDNA3.1 vector map [file BAB-72-1528-s001.tif]

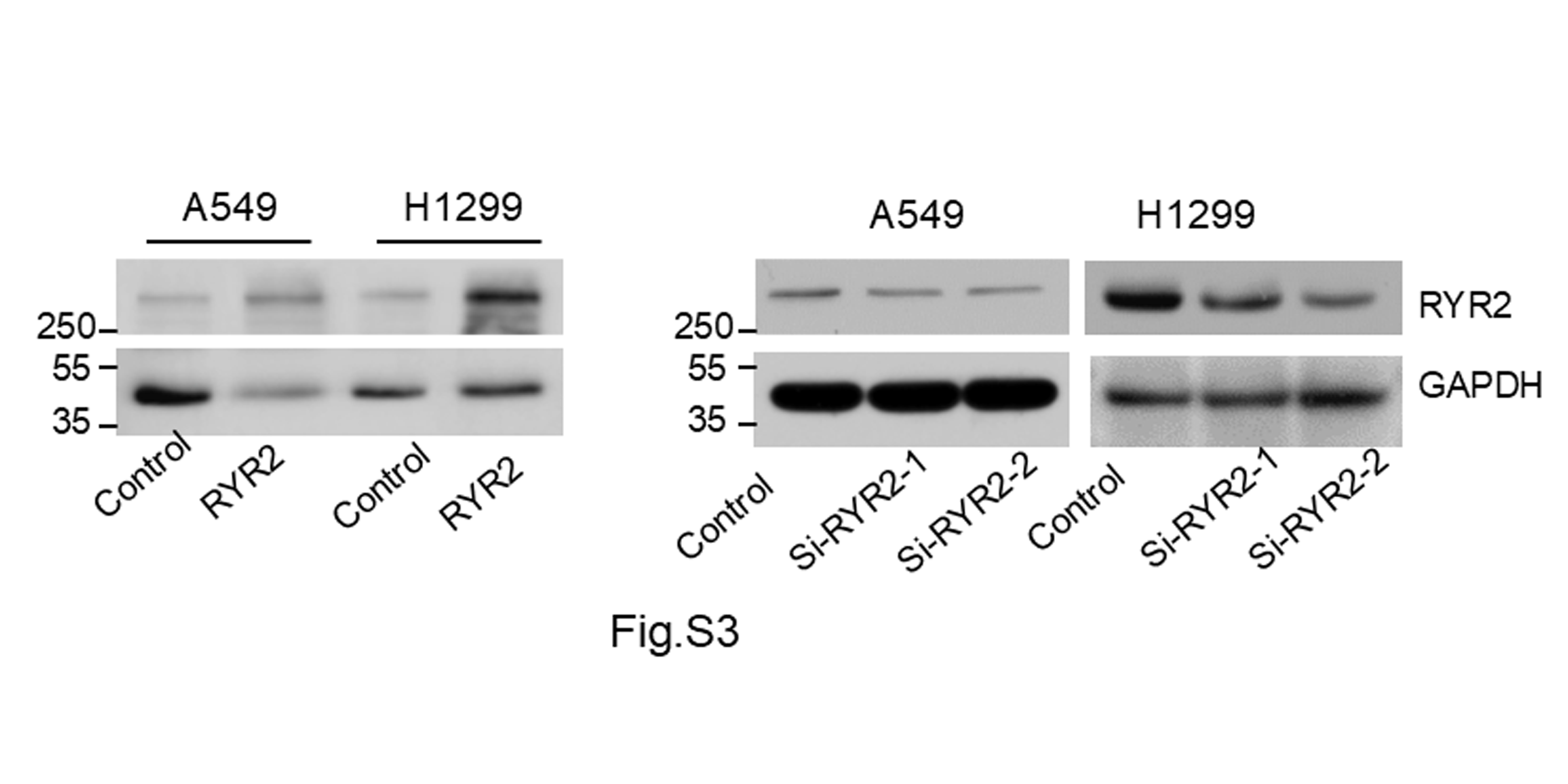

Supplement: Supplementary file 3 — Figure S3. RYR2 overexpression or knockdown in LUAD cells. RYR2 overexpression vector or specific siRNAs targeting RYR2 were transiently transfected into A549 and H1299 cells. The RYR2 level was detected by immunoblotting. [file BAB-72-1528-s003.tif]
